# The Iron Metabolism with a Specific Focus on the Functioning of the Nervous System

**DOI:** 10.3390/biomedicines12030595

**Published:** 2024-03-06

**Authors:** Monika Kulaszyńska, Sebastian Kwiatkowski, Karolina Skonieczna-Żydecka

**Affiliations:** Department of Biochemical Science, Pomeranian Medical University in Szczecin, Broniewskiego 24, 71-460 Szczecin, Poland; sebastian.kwiatkowski@stmedical.clinic (S.K.); karzyd@pum.edu.pl (K.S.-Ż.)

**Keywords:** iron, brain, iron metabolism, hepcidin

## Abstract

Iron is the micronutrient with the best-studied biological functions. It is widely distributed in nature, and its involvement in the main metabolic pathways determines the great importance of this metal for all organisms. Iron is required for cellular respiration and various biochemical processes that ensure the proper functioning of cells and organs in the human body, including the brain. Iron also plays an important role in the production of free radicals, which can be beneficial or harmful to cells under various conditions. Reviews of iron metabolism and its regulation can be found in the literature, and further advances in understanding the molecular basis of iron metabolism are being made every year. The aim of this review is to systematise the available data on the role of iron in the function of the nervous system, especially in the brain. The review summarises recent views on iron metabolism and its regulatory mechanisms in humans, including the essential action of hepcidin. Special attention is given to the mechanisms of iron absorption in the small intestine and the purpose of this small but critically important pool of iron in the brain.

## 1. Introduction

Iron is the most abundant metal in nature. Predominantly, iron is an essential cofactor of many cellular enzymes [1]. In humans, the daily requirement for this element is approximately 25–30 mg, depending on age, lifestyle, and gender. In paralel, iron deficiency is one of the most common single nutrient deficiencies [2,3]. Iron is essential for the general vital functions of every cell, as well as for certain neuronal activities. Excessive iron levels promote oxidative stress and mitochondrial damage, leading to cell death, including neuronal death. Neurodegeneration can therefore be defined by changes in ionic homeostasis and/or pro-oxidant/antioxidant balance, two states that vary significantly between different types of brain cells and with age [4]. Iron plays an important role in brain development from the prenatal period to the teenage years. The blood–brain barrier (BBB) modulates iron levels in the brain. In case of iron deficiency in the child, negative effects on myelinogenesis and synaptogenesis have been demonstrated, with negative effects on psychomotor and cognitive functions [5].

The evidence clearly indicates that abnormal iron levels may contribute to the development of diseases such as multiple sclerosis (MS), Parkinson’s disease (PD), Alzheimer’s disease (AD), or other neurodegenerative diseases [6]. Iron homeostasis is the absorption, storage and excretion, all of which require comprehensive control [1]. A system in which iron enters the brain by regulated receptor-mediated transport and exists via mass flow is ideal for iron homeostasis in the brain [7]. The brain has a high metabolic activity and actively controls the maintenance of the continuity of biological processes, ensuring the balance of iron metabolism within its structures and throughout the body [8].

## 2. Iron Metabolism in the Brain

Iron turnover in the brain begins at the BBB. It is absorbed by cerebrospinal fluid (CSF) or by primary binding to transferrin (TF) and, as such, crosses the BBB. These processes efficiently regulate the flow and prevent excessive accumulation of this element [9]. Iron turnover in the brain is much slower compared to other organs [10]. Three cell types are responsible for coordinating this: brain microvascular endothelial cells (BMVECs), astrocytes, and pericytes, with the latter ones likely responsible for vasoconstriction and vasodilation [11].

Humans obtain iron from different food sources and supplements. However, the majority comes from dead red blood cells. Dietary iron, once absorbed and further released from the enterocytes, is oxidised and immediately binds to TF. TF is a protein carrier that binds two iron atoms (Fe^3+^) with high affinity [12]. TF distributes iron throughout the body, to all cells, including BMVECs. Its role is to mediates between iron storage, absorption, and utilisation [13].

The resulting TF–iron complex circulates in the blood and eventually binds to the transferrin receptor (TFR) in target cells, preferably in an inert environment [14,15]. At the BBB level, in the process of iron uptake, the first cells to come into contact with the TF–iron complex are BMVECs [16]. They have an analogous structure to the absorptive enterocytes through which iron is taken up from the intestine into the blood [12]. As in the intestinal barrier, tight junctions are located between BMVECs, which block the entry of iron via intercellular pathways and create the best transcellular route for it [16].

Much of the control of brain iron uptake by BMVECs lies in the regulation of the levels of transferrin receptors 1 and 2 (TFR1 and TFR2) at the BBB [9,17]. Their presence on the surface of BMVECs allows the complex to be taken up by an endocytic process [18,19]. TFR1, the major receptor for TF, is abundant throughout the nervous system, particularly in neurons [20]. TFR2 has a lower affinity for TF than TFR1 and is mainly found in the mitochondria of dopaminergic neurons. Unlike TFR1, is not controlled by the intracellular iron level as it has no iron-sensitive elements [21]. Iron transport by BMVEC can be reduced by blocking the TFR with specific antibodies [22].

TF–iron and TFR form an endosome inside which, due to the acidic pH, the complex is degraded. The next step is the reduction of iron (Fe^3+^) to a more soluble form, i.e., Fe^2+^, and its release into the cytoplasm via the divalent metal ion transporter 1 (DMT1) [16]. There are four isoforms of DMT1, two of which contain an iron-responsive element (IRE) in the last exon of corresponding gene [18]. IREs are responsible for controlling iron homeostasis through the regulation of ferritin, ferroportin (FPN), and TFR [23]. DMT1 is also responsible for the uptake of non-transferrin-bound iron (NTBI) into BMVECs by importing it onto the lumen surfaces of BMVECs [16]. Prior to this, NTBI may enter the brain via the epithelial cells of the choroid plexus. It is probable that Fe^3+^ is first reduced to Fe^2+^ by cellular reductases like ascorbate, followed by transportation using DMT1 [24]. According to Ji et al. NTBI transportation in brain cells is also dependent on proteins such as Zip8 and Steap2, although their precise role is not yet wholly understood [25]. Both iron sources exit the BMVEC through the ferrous iron transporter, ferroportin (FPN) [16,17].

The cytoplasm is the site where iron is taken up and used for metabolic processes, such as the synthesis of haem groups or iron–sulphur centres. Excess can be stored in neuromelanin (NM) or ferritin, the latter one being the main protein responsible for storing iron in a harmless form and delivering it in to the appropriate cells in sufficient quantities [12].

Ferritin consists of two subunits, L and H, which form a cavity of 24 subunits that can store approximately 4500 iron atoms. The H-ferritin subunit has its own iron oxidation site, which allows for faster initial iron uptake [26]. The H-subunit is mainly found in neurons, while the L-subunit is found in astro- and microglia, with both forms being equally abundant in oligodendrocytes. NM traps large amounts of iron in some neuronal structures and is involved in its long-term storage. The free form of iron is toxic to the body due to variation in oxidoreductive potential and catalysis of free radical reactions. Ferritin and NM counteract these damaging reactions [27]. After iron leaves the cell with the FPN exporter, two enzymes become active. The release of the element outside the cell depends on the iron-oxidazing activity of ceruloplasmin (CP) or hephaestin (HP); both enzymes belong to the group of ferroxidases and require copper ions for their correct functioning [28]. The main function of CP is the oxidation of ferrous ions (Fe^2+^) to ferric ions (Fe^3+^), which enables the binding of iron to TF and ferritin. It has been demonstrated that the main source of CP in the brain are astrocytes. Astrocytesproduce a CP linked with glycosylphosphatidylinositol (GPI-Cp). This form of CP plays a role in iron metabolism in the brain. Consequently, if the CNS is not functioning properly, the process of iron oxidation does not occur with sufficient efficiency, as it has been shown that the level of GPI-Cp is reduced. As a result, the amount of Fe^3+^ ions that can be bound by transferrin decreases, while the amount of Fe^2+^ unbound by transferrin increases. The lack of GPI-Cp in the CNS also leads to an accumulation of Fe^2+^ ions in the cells, as ferritin can only bind Fe^3+^. This leads to conditions of oxidative stress [15].

The distribution of iron from BMVEC to brain cells is not entirely clear, but it is very efficient in that it does not significantly reduce brain iron levels, even in the presence of systemic iron deficiency [29]. Astrocytes, according to in vivo studies, are key mediators of iron transport from BMVECs to brain cells [28]. First, they secrete ferroxidases, which normalise the BMVEC-FPN complex. They are also a source of hepcidin that controls the influx of iron into the brain and local regulators of neuronal homeostasis during iron overload [30,31,32]. Once iron crosses the BBB and enters neurons via the TFR and DMT1, it is used there for metabolic purposes [33]. These two proteins (TFR and DMT1) show higher expression in neurons than in glial cells [34].

### Regulation of Iron Metabolism in Humans

The correct amount of iron is required for various molecular processes such as energy production and haem formation. Too much iron can be harmful by inducing the formation of reactive oxygen species, which cause oxidative stress, resulting in DNA damage, increased lipid peroxidation, and cellular ferroptosis. Furthermore, even if we have adequate iron stores, abnormal iron distribution can lead to anaemia. While iron loss is fairly constant and unregulated, the uptake and release of iron from cells depends on a number of proteins. This allows intracellular and systemic iron levels to be maintained at adequate and safe levels [35]. Iron metabolism in the CNS is coordinated by two regulatory systems. The first controls iron metabolism at the cellular level through post-transcriptional regulation of iron-regulatory proteins, and the second operates at the systemic level through hepcidin, the hormone that regulates ferroportin (FPN) expression [36].

Post-transcriptional regulation of cellular iron metabolism relies on the action of two iron regulatory proteins: IRP1 and IRP2. They bind iron responsive element (IREs), which are stem-loop structures located in the 3′UTR and 5′UTR regions of mRNAs of proteins responsible for iron homeostasis. In case ofiron deficiency, IRP interacts with IREs in the 5′UTR of ferritin or ferroportin mRNA sequences, resulting in the inhibition of the synthesis of these proteins [37]. This reduces unnecessary storage and excretion and increases free iron in the cells [38]. Binding at the 3′UTR of TFR or DMT1 mRNAs increases their synthesis and promotes iron absorption [36]. In contrast, the system-wide mechanism for controlling iron homeostasis is based on the action of the protein hormone HAMP [35].

Collected data from various studies indicate that hepcidin (encoded by the *HAMP* gene) is a key regulator in maintaining iron homeostasis. Once hepcidin is expressed, iron intestinal absorption is inhibited, and decreased recycling by macrophages and mobilization from liver stores take place [39,40,41]. In the case of hepcidin, this mainly involves controlling *FPN* expression at its translational stage [9]. When iron levels are too high, HAMP on the surface of lymphatic enterocytes, macrophages, hepatocytes, and placental cells binds FPN and phosphorylates its tyrosine residues, leading to its lysosomal degradation. FPN is the only exporter of iron from the cell, so inhibition of its expression will ultimately lead to accumulation of iron [42,43,44,45]. This mechanism results in reduced absorption of iron from food, less efflux of recovered iron from macrophages, and less consumption of iron stored in liver cells [46,47]. The exact opposite happens during iron deficiency. This was confirmed in in vivo studies with iron-deficient rats manifesting high decreases in *HAMP* gene expression [48,49]. The study demonstrates not only an inhibitory effect on iron export but also on import through DMT1 and TFR1 [48,50]. Du et al. conducted a study to see what effect hepcidin has on the expression of iron uptake proteins (TFR1, DMT1, and FPN) in cultured astrocytes. The results showed that astrocytes treated with hepcidin peptide had a strongly reduced ability to take up iron but also to release it, and the mechanism responsible for this was a reduced expression of iron transport proteins [51]. There are other studies also showing a regulatory role for hepcidin in the brain [52,53]. HAMP mRNA levels in different brain areas increase with age [54], and administration of hepcidin by injection into the lateral ventricle of the brain reduced the influx of iron into the brain tissue and attenuated the brain overload with iron [55].

Inflammation is a definite inducer of hepcidin expression in the brain—up to a 40-fold increase in the case of *E. coli* infection [56]. If a bacterial lipopolysaccharide (LPS) is administered to choroid plexus epithelial cells, the level of interleukin-6 (IL-6) elevates, resulting in the expected increase in signal transducer and activator of transcription 3 (STAT3) pathway levels. This ultimately results in an increase in *HAMP* expression [57]. LPS and IL-6 have also been linked to an increase in *HAMP* expression in brain parenchyma [50,56,58], astrocytes, and probably neurons [59,60] and microglia [61,62]. Urrutia et al. conducted a study using primary cultures of astrocytes and microglia treated with Aβ and hepcidin. The authors then assessed cytokine levels in the cultures, tested the toxicity of media conditioned with astrocytes or microglia, and finally assessed cell death and oxidative stress generation [63]. They found that HAMP attenuated inflammatory and pro-oxidative processes induced by β-amyloid (Aβ) in astrocytes and microglia and thus protected neighbouring neurons from damage [63,64]. Further evidence for the anti-inflammatory effect of hepcidin comes from a study by De Domenico et al. The study proved that following HAMP administration, LPS-induced cytokine synthesis was reduced [65]. Interestingly in studies using higher levels of hepcidin, the efficacy of malaria treatment was elevated [66]. The anti-inflammatory function of hepcidin may serve to close the feedback loop controlling the inflammatory response, and it is of sufficient interest to warrant further investigation [9].

## 3. Iron Functions in the Brain

**Figure 1:**
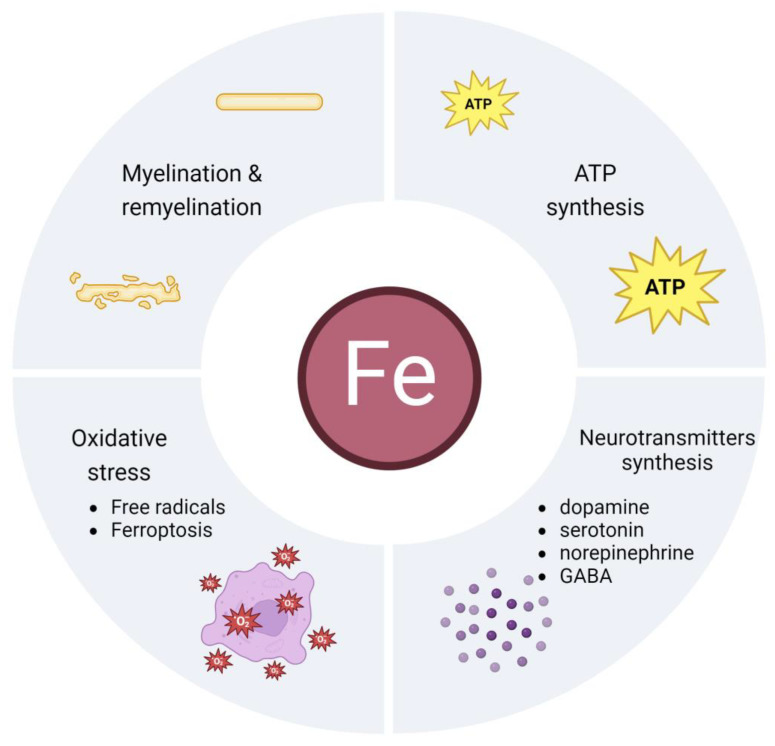
Iron functions in the brain.

Of the total pool of iron contained in the human body, only 2% is used in the brain. On the other hand, the proper management of this relatively small amount controls the proper functioning of the human body and vice versa. A number of key processes in which this biometal is involved originate in the brain. Iron performs its function by being part of enzymes in the form of its prosthetic group as iron–sulphur or haem centres. It is a component of a very large number of proteins and enzymes for almost all living organisms. The processes in which it is involved precisely as a prosthetic group are the transport of electrons and oxygen in the respiratory chain, gene expression, DNA synthesis and repair, the production of neurotransmitters and myelin, and the facilitation of chemical reactions by binding to the substrate of enzymes [67,68]. Also, the cells of our body contain a large number of proteins that require iron for their structure and function, such as haemoproteins or ribonucleotide reductases (RNRs). The latter ones use iron as a cofactor to fulfil its tasks of DNA replication and DNA repair [69]. Iron functions in the nervous system are illustrated in Figure 1 and discussed in more detail later in this review.

### 3.1. Oxidative Stress

Iron metabolism underpins the dynamic interplay between oxidative stress and antioxidants in several processes. Both its deficiency and excess affect the redox state. Maintaining normal levels of this element is essential for the proper functioning of the brain and the body as a whole [70]. Iron can also be a toxic molecule due to its ability to accept and donate electrons. Its most toxic property is its involvement in catalysing the formation of free radicals from reactive oxygen species (ROS) in the Fenton reaction. This reaction involves the reduction of H2O2 by a single electron, and the result of this process is the formation of a hydroxyl radical (۰OH) which, when in excess, causes damage to many cellular structures [71]. Therefore, most of the iron is bound to different molecules and stored or transported, and only a small amount remains unbound [36]. Transferrin increases the solubility of ferrous ions (Fe^3+^) and transports them to structures that need the iron. Excess iron in the cytosol is also retained by ferritin, but sometimes the amount exceeds its capacity. Free iron is the cause of toxic effects on the cell and the production of ROS. Hence, it is inevitable in the body to form ROS, which increase the permeability of the mitochondrial membrane, damage the lysosomal membrane, and cause iron to escape into the cytosol [72]. ROS generate reactive aldehydes, which, along with further oxidative damage mediators, cause oxidative modification of proteins, manifesting as carbonyl compound formation. These misfolded and damaged proteins cannot be degraded by the ubiquitin/proteasome system and accumulate as the characteristic inclusion bodies seen in many neurodegenerative diseases. Additionally, ROS have the ability to degenerate nucleic acids, leading to faster ageing, cancer formation, and the aforementioned neurodegenerative diseases [73,74]. Fortunately, the human body has detoxifying capacities to regulate the production of ROS and restorative capacities to repair the damage caused by ROS [75]. The CNS is more susceptible to oxidative stress because catalase, superoxide dismutase, and glutathione peroxidase, the enzymes involved in scavenging free radicals, are less active in the brain [76]. Iron also acts as a cofactor for tyrosine hydroxylase, an enzyme involved in the synthesis of dopamine, which is essential for maintaining neuronal function and normal viability [77].

Oxidative cell death due to iron ion accumulation called ferroptosis has also been identified. Features of ferroptosis include an increased intracellular pool of unbound iron, increased lipid peroxidation in the cell membrane, and depletion of reduced nicotinamide adenine dinucleotide phosphate (NADPH) [70]. This causes the mitochondria to shrink and increases the density of their membranes. Iron accumulation then leads to an increase in cytotoxic lipid ROS in the cell. This can be prevented by iron chelation, such as with deferoxamine, which removes excess iron ions from the cells, or by using a lipophilic antioxidant [78]. Iron interacts with dopamine to produce 6-hydroxydopamine quinone (6-OHQD), which, in turn, reacts with glutathione peroxidase 4 (GPx4) to cause ferroptotic cell death. It is concluded that ferroptosis is probably the cause of dopaminergic neuronal death and leads to the development of neurodegenerative diseases [79].

During an infection or inflammation, the body absorbs smaller amounts of iron in order to deprive the pathogenic microorganisms of this element which they need to bloom. This iron deficiency can lead to oxidative stress [80]. Chronic inflammation caused by cancer or inflammatory diseases decreases *HAMP* expression, resulting in reduced intestinal iron absorption and iron retention in macrophages [80,81]. Oxidative stress also occurs in people with chronic iron-deficiency diseases, linked to the negative regulation of ferroportin by hepcidin [82]. The anaemia-induced hypoxic state can worsen oxidative stress through altered cellular metabolism, increased catecholamine metabolism, and leukocyte activation, resulting in increased ROS production [83].

### 3.2. Production of ATP

Another very important function of iron is the production of adenosine triphosphate (ATP) in the mitochondria. Iron is a cofactor for cytochromes and iron–sulphur complexes in the respiratory chain. Glucose is the main substrate for energy production in the brain. It has been shown that 20% of the whole body’s energy is used by the brain, even though it makes up for only 2% of its mass. Approximately 80% of energy is used to support neurons and the remaining 20% is used to maintain the function of astrocytes, microglia, and oligodendrocytes [84]. Neurons use energy to send synaptic and axonal signals, as well as postsynaptic signals [85]. Oxidative phosphorylation takes place in the mitochondria, and large amounts of energy are produced at the end of this process. The main mechanism of the respiratory chain is based on the transfer of electrons from nicotinamide adenine dinucleotide (NADH) and flavin adenine dinucleotide (FADH_2_) to the acceptor oxygen [86]. The respiratory chain is made up of four large protein complexes. The complexes contain either iron and sulphur clusters (ISCs) or haem-containing proteins in their structure. Complex I (NADH dehydrogenase) carries eight ISCs, complex II (succinate dehydrogenase) carries three ISCs and one haem protein, complex III (cytochrome C oxidoreductase, cytochrome bc1) holds one ISC and several haem groups, and complex IV (cytochrome C oxidase) has two haem residues. Enzymes involved in the tricarboxylic acid (TCA) cycle also contain ISCs. One such example is aconitase. Iron acts as a cofactor for these proteins by accepting electrons, oscillating between the Fe^2+^ and Fe^3+^ states, and transporting them up the respiratory chain using its redox properties [84].

Cytochrome C oxidase (CytOx) is the terminal iron enzyme in the respiratory chain, and its activity reflects neuronal metabolism. Studies in newborn rats have shown that perinatal iron deficiency reduces neuronal activity, particularly evident in brain areas involved in memory processing [86].

In summary, as iron is a basic building block of many respiratory chain proteins involved in electron transport in the respiratory chain, the presence of iron is essential to produce the required amounts of ATP. It is therefore very important that the mitochondria are supplied with enough iron to keep these processes going [84].

### 3.3. Processes of Myelination and Remyelination

Myelin is the substance that forms the sheath of nerve fibres and insulates the transmission of electrochemical signals along axons. Its function is to transmit information to muscles, glands, and nerve cells. Myelin is produced by the cells surrounding the axons and is thought to nourish them, speed up transmission, and improve neural circuits. It is produced in the central nervous system (CNS) by oligodendrocytes (OL) and in the peripheral nervous system by Schwann cells (SC) [87]. OL are the major cells of the CNS that stain for iron under physiological conditions [88]. Myelination involves the spiral wrapping of plasma membranes around axons [87]. This process in the brain is essential for the normal development of its cognitive, sensory, and motor functions [89].

Studies have shown that reduced dietary iron availability promotes hypomyelination. The timing of iron delivery to the OL during development is itself very important, as hypomyelination and associated neurological complications persist long after systemic iron deficiency has been corrected [88]. Iron deficiency affects the proliferation of oligodendrocyte precursor cells (OPCs), resulting in reduction in the number of oligodendrocytes produced [90]. Limited iron supply during pregnancy and the postnatal period reduces the amount and composition of myelin [84]. During hypoxia, OLs can accumulate more iron, leading to stress on the endoplasmic reticulum, abnormal protein folding, and production of reactive oxygen species, which ultimately lead to myelination deficits [89]. A histopathological study was carried out on the brains of newborn rats in two groups: iron-deficient and iron-supplemented. The first group showed reduced myelination in the spinal cord and white matter of the cerebellar folds [91]. Iron-deficient infants were also studied and followed from preschool to adolescence, and inferior neurological characteristics such as poorer cognitive, motor, and socioemotional functions and neurophysiological differences were documented in these infants [90].

Oligodendrocytes require large amounts of ATP to perform their functions and thus require a constant supply of iron. Cholesterol and fatty acids make up about 70% of the dry weight of myelin. Cholesterol and fatty acid synthesis pathways are also iron-dependent, thus essential for myelination. There are enzymes involved in these pathways in OL, and iron is their cofactor [84]. Although transferrin is an important substrate component in OL culture, in vivo OLs do not have transferrin receptors. In contrast, OLs have specific receptors for ferritin H (HF) and the uptake of extracellular HF by oligodendrocyte precursors (OPCs) occurs via endocytosis. These data demonstrate that ferritin is the major source of iron for OL [88].

The process of remyelination is also influenced by iron. Loss of OL leads to demyelination, the loss of myelin sheaths on axons. And it is remyelination that is a process that is spontaneously triggered in such circumstances and is very effective in regenerating myelin sheaths in exposed axons [92]. Remyelination in the CNS is carried out by OPC cells, which invade sites of myelin defects then proliferate and transform into OLs, regenerating the damaged areas with newly formed myelin [93]. There are a number of growth factors that regulate the proliferation and transformation of OPCs, and these include fibroblast growth factor 2 (FGF-2) and insulin-like growth factor 1 (IGF-1) [94]. This regulation is also controlled by cytokines such as tumour necrosis factor-α (TNF-α) [95] and interleukin-1β (IL-1β) [96].

Iron is thought to enter the CNS from the circulation via capillary endothelial cells [19]. Approximately 95% of the capillary surface is covered by astrocytes, making them ideally placed to take up iron from the circulation and distribute it to other CNS cells [31]. Indeed, astrocytes have the iron influx and efflux mechanisms necessary to transport this element from cell to cell, namely, the iron exporter ferroportin discussed earlier. In addition to the direct effects of iron deficiency on OPCs, iron also has indirect effects on remyelination, such as altering the levels of cytokines and growth factors that can influence OPC proliferation and differentiation [93].

### 3.4. Neurotransmitter Synthesis and Metabolism

Neurotransmitters bind to second messenger-linked receptors to initiate a complex cascade of chemical events that can either excite or inhibit further electrical signals. The complete process of this communication between cells involves the synthesis, transport, storage, control of release, and binding of these neurotransmitters to neuronal receptors. Iron plays a role in each of these processes [84]. Monoamine neurotransmitters include dopamine (DA), serotonin (5-HT), or norepinephrine (NA), which are involved in the regulation of cognitive, emotional, and excitatory processes. Another type of neurotransmitter is gamma-aminobutyric acid (GABA), which has a calming effect in the CNS [97]. Serotonin is synthesised by tryptophan hydroxylase (TPH), and dopamine and noradrenaline by tyrosine hydroxylase (TH) [98], whereas gamma-aminobutyric acid is synthesised by glutamate dehydrogenase and GABA transaminase [99]. Both hydroxylases are homotetramers using non-haem iron as their cofactor to catalyse the reaction to incorporate one molecular oxygen atom into the substrate, leading to the formation of hydroxylated products. In the absence of iron, all three enzymes cannot perform their function, resulting in inhibition of the synthesis of these neurotransmitters [100].

A study has been carried out indicating that the conversion of dopamine to norepinephrine in the brain is impaired in iron deficiency. This was manifested by a reduced function of monoamine transporters and receptors [101]. An experiment by Erikson et al. showed abnormal density and function of dopamine receptors in several brain areas due to regional iron loss [102]. Similar conclusions have been drawn from other studies, where the activity of dopamine-containing brain areas and the density of dopamine receptors were shown to depend on iron concentration [103]. A very prominent feature of iron deficiency is a reduction in dopamine neurotransmission resulting from a severely reduced number of dopamine D2 receptors in the brain. The consequences of reduced dopaminergic neurotransmission aew changes in dopamine-dependent behaviour, the most important of which is a reduction in learning processes and thus cognitive functions. Disruption of iron metabolism in young adulthood may cause irreversible damage to dopamine neurons, which may not become apparent until adulthood [104]. In the case of serotonin, the rate-determining enzyme for the synthesis of this neurotransmitter, TPH, can be inhibited by iron chelators [100]. There is a study [99] showing the effect of iron also on GABA, which describes how iron deficiency impairs the activity of two enzymes responsible for GABA synthesis, namely, glutamate dehydrogenase and GABA transaminase. After a week supply of dietary iron, the reduced activity of these enzymes returned to appropriate levels. Long-term iron deficiency resulting in reduced GABA production can lead to endocrine and neurological disruptions, along with behavioural alterations [99]. An important aspect in the context of limiting the effects of iron deficiency on neurotransmitter synthesis is the timing of iron supplementation. When treatment of iron-deficient rats was undertaken on postnatal day 4, iron deficiencies could be replenished and subsequent consequences prevented; this contrasts with treatment undertaken on postnatal day 7 and beyond, where making up for losses became impossible [105].

## 4. Iron in Brain Development and Brain Diseases

### 4.1. Brain Development

The very first 1000 days of life—from conception to the second birthday of a child—are crucial for proper central nervous system development [106]. During this period, especially during pregnancy, the host’s requirement for iron is up to 10 times higher compared to the non-pregnant period, as this element is needed to meet maternal iron requirements and to support fetal and placental development [107]. Later, after birth, the demands for iron remain high [108]. Importantly, iron deficiency in pregnancy accounts for those of up to 65% of women [109,110,111], even though during this period, some physiological adaptations (hepcidin) occur to facilitate elevated iron absorption and further metabolism. Up to around 4–6 months of age, the storage of iron seems to be sufficient even with exclusive breastfeeding [112]. After this period, with the introduction of the solid foods into the child’s diet, the requirement for iron supply from external sources increases [113]. Sadly, the iron deficiency is prevalent among European children [114]. The consequences of an iron deficiency during the first 1000 days of life are detrimental to the developing brain. The disruption of processes such as myelination and neurotransmitter signalling, as well as energy metabolism, has been well documented. These can further impair the development of the visual and auditory cortex, receptive language and speech production, and finally, higher cognitive functions [115,116]. Iron deficiency anaemia in pregnancy has been linked to higher odds for autism spectrum disorders and attention-deficit/hyperactivity disorder in the offspring [117]. Additionally, the long-term effects may also be detrimental, including poor recognition memory and impaired motor skills in childhood [118] and behavioural disturbances in adulthood [119]. Much effort in health policy must be taken on preventative measures, as iron supplementation was found to improve intelligence, attention, concentration, and memory in school-aged children [120].

### 4.2. Neurodegenerative Diseases

There is evidence to support a conclusion that imbalanced iron metabolism may be involved, among others, in the occurrence of neurodegenerative diseases [121,122]. It is very important to maintain iron levels at an appropriate level, and a good way to reduce iron accumulation is the use of iron chelators, which can cross the BBB and reduce iron accumulation, thus providing neuroprotection [68]. Iron accumulation in the brain can cause free radical formation through the Fenton reaction, and this promotes diseases such as Parkinson’s disease (PD), Alzheimer’s disease (AD), multiple sclerosis (MS), or other neurodegenerative disorders [121,122]. To add, in several neurodegenerative diseases of genetic origin, decreased expression of proteins that directly affect iron metabolism has been observed. These diseases include Friedreich’s ataxia, aceruloplasminemia, neuroferritinopathy, and Huntington’s disease [123,124,125].

The association of iron with mechanisms of nervous system disease has been confirmed in various studies [126,127]. Abnormalities in redox-mediated iron homeostasis are implicated in AD neuropathology. Magnetic resonance imaging (MRI) that was used to determine iron shows that brain iron levels in AD patients increase with age [128]. Another analysis, such as inductively coupled plasma mass spectrometry (ICP-MS), has shown that plasma iron levels decrease in Alzheimer’s patients, presumably due to increased brain iron [129]. Patients with preclinical Alzheimer’s disease had increased iron levels and iron redox activity in the cerebral cortex and cerebellum [128]. Iron regulation in AD pathogenesis involves the process of abnormal folding of amyloid (Aβ), amyloid precursor protein (APP) and hyperphosphorylated tau, resulting in oxidative stress to neurons [130].

Of particular interest in the role of iron is the area of the brain most affected by Parkinson’s disease. The “black substance” derives its name from the oxidation reaction between dopamine, a neurotransmitter, and iron, which is abundant in the nucleus accumbens of neurons, resulting in the formation of an insoluble black substance known as neuromelanin. Iron accumulates in this structure with age under normal conditions, but at a higher rate in PD. Neuropathological studies using spectroscopic methods to measure the total concentration of iron in neuromelanin show that it increases with the severity of PD [131,132]. Reasons attributed to brain iron accumulation in PD patients include increased expression of DMT1 in dopamine neurons [18], altered iron transport by type 2 transferrin [133], and mutations in genes responsible for iron transport and binding [134,135]. Studies show an increase in redox-active iron bound to neuromelanin in the black matter neurons of PD patients, probably due to a decrease in ferritin synthesis. Iron levels are higher in patients with the greatest neuronal loss and are absent in neurons without neuromelanin [136,137]. Additionally, the total iron content was examined via MRI in the red nuclei of PD patients and found to be at excessively high levels [138].

In MS, increased levels of iron are found in specific areas of the brain, such as deep grey matter structures and white matter. As with Parkinson’s disease, iron levels in the brain of people with MS increase as the disease progresses [139]. How iron accumulates in these structures is incompletely understood, but there is evidence that it is related to inflammatory processes that increase the permeability of the BBB and allow iron-rich macrophages to enter the brain [140]. Inflammatory processes in MS include the activation of microglia and the release of pro-inflammatory cytokines and ROS, which induce oxidative stress [141]. This inflammatory environment can cause excessive degradation of oligodendrocytes, resulting in the release of additional redox-active iron into the brain, further increasing oxidative stress [140].

Prion diseases, such as Creutzfeldt–Jakob disease (CJD), are also fatal neurodegenerative diseases, involving the conformational conversion of a normal cellular prion protein (PrP^c^) into a pathogenic one (PrP^sc^), which tends to form aggregates and amyloid fibrils, which, in turn, promote the abnormal folding and aggregation of normal PrP^c^ [142]. This process renders PrP^Sc^ insoluble in non-ionic detergents and confers limited protease resistance, significantly increasing its half-life [143]. PrP^Sc^ aggregates in the brain parenchyma contribute to disease-related neurotoxicity. The conformational change from PrP^c^ to PrP^Sc^ occurs reflexively in spontaneous disease. In contrast, it is facilitated by mutations in the *PrP^c^* gene in familial disease and can also result from direct exposure to an exogenous source of PrP^Sc^ in infectious disease [143,144]. To maintain normal iron homeostasis, it is important to understand the physiological and pathological interactions of PrP^c^ and PrP^Sc^ with iron and the contribution of these processes to the pathogenesis of prion diseases [145]. There is increasing evidence that PrP is involved in iron homeostasis. Mice lacking *PrP* gene show an altered iron metabolism and reduced brain iron levels [146]. PrP^Sc^ and ferritin complex is of high redox activity and is highly cytotoxic unless rapidly degraded by cellular mechanisms [147,148]. Co-aggregation of ferritin with PrP^Sc^ results in the sequestration of bound iron into inaccessible forms, leading to a cellular iron deficiency phenotype. Elements indicative of this include increased expression of the iron uptake proteins TF and TFR and decreased levels of cellular ferritin to reduce iron storage [149]. Additionally, patients with CJD have altered levels of ferroxidase and transferrin in the CSF [150]. PrP^c^ models cellular iron uptake and induces the conversion of Fe^3+^ to Fe^2+^ [146,151]. PrP^c^ gene mRNAs contain IREs that control iron homeostasis. The binding of IRP proteins to IREs is influenced by changes in intracellular iron levels. In the absence of iron, IRE/IRP complexes increase the stability of transferrin receptor mRNA and stimulate cells to import iron [152].

### 4.3. Brain Tumours

There is a body of evidence linking altered iron metabolism and brain tumour development [153], especially as this element is crucial for cell death—a cell cycle phase being the focused of translational research [154]. Indeed, the cells of a tumour divide more rapidly in comparison to normal cells; thus, their demand for iron is higher. Upregulation of genes that play a critical role in iron metabolism—including TFR1 and STEAP3, the latter of which is both an iron transporter and an inhibitor of cell apoptosis [155]—has been confirmed in some types of cancer. As a result, oxidative stress in the tumour microenvironment is high, and tumour cells upregulate antioxidant pathway genes to sustain their growth [153,156]. 

Some iron metabolism factors have been linked to modulating tumour progression; among them is IRP2 regulated by ubiquitin ligase FBXL5, with the latter increasing iron levels and producing [2Fe2S] clusters to further promote IRP2 polyubiquitination and degradation in response to iron and oxygen concentrations [157]. Also, FPN1, the iron export protein, has been found to be downregulated in some tumours [153]. Importantly, Geng et al. found that in the FPN1^−/−^ model, neuroblastoma suppression was enhanced by increasing ROS synthesis [158]. Further studies are required to determine the roles of alterations in iron metabolism genes in brain tumours during cancer progression. To add, a known mechanism of ferroptosis has been reviewed in a few brain tumour studies [159]. When rats transplanted with glioma-35 cells were given iron-supplemented water and chelating desferroxamine prior to radiotherapy, glioma growth was promoted, but the efficacy of radiotherapy was improved by apoptosis and ferroptosis. It has been shown that neutrophils infiltrating the tumour tissue may enhance lipid peroxidase with the appearance of myeloperoxidase [160]. In dopaminergic neuroblastoma, light has been shed on the overexpression of mitochondrial ferritin (FtMt), which was found to inhibit the cellular labile iron pool (LIP), which further causes the accumulation of ROS, protecting from ferroptosis effects [161]. Recently, the expression of Merlin/Neurofibromin2 (NF2) and the ferroptosis regulator GPX4 were positively correlated in the case of primary meningioma. The authors stated that the lower the expression of *NF2* and its transcription factor *MEF2C*, the higher the odds for ferroptosis; thus, lower the odds for meningoma growth [162].

## 5. Conclusions

There is a growing interest in maintaining normal iron homeostasis in the brain in fields such as neurology and neuropharmacology. A thorough understanding of the mechanisms involved in iron homeostasis is essential to explain the pathological responses that lead to excessive iron accumulation in the brain. Elucidating the mechanism of these responses will help in the development of pharmacological interventions that can break the chain of abnormal events that occur in neurodegenerative diseases, especially those caused by iron accumulation. Understanding the details of iron regulation in the brain will help prevent neuronal death, which is the initial cause of diseases such as MS, PD, and AD. The information presented in this article on the many important functions of iron in the nervous system clearly implies that therapeutic efforts should be directed towards learning how to maintain normal iron homeostasis, to inhibit the production of ROS, and to prevent oxidative stress. There is an urgent need to improve our understanding of intestinal iron absorption and its relationship to iron balance in our body’s cells. Not all genes involved in iron transport and iron metabolism have yet been identified. A better understanding of these all-important aspects will improve our knowledge of, and our ability to manage, iron deficiency and iron overload.
